# Immune Checkpoint Inhibitors in Hepatocellular Carcinoma: Current Progresses and Challenges

**DOI:** 10.3389/fonc.2021.737497

**Published:** 2021-10-22

**Authors:** Hao-Tian Liu, Meng-Jie Jiang, Zhu-Jian Deng, Le Li, Jian-Li Huang, Zhen-Xiu Liu, Le-Qun Li, Jian-Hong Zhong

**Affiliations:** Hepatobiliary Surgery Department, Guangxi Liver Cancer Diagnosis and Treatment Engineering and Technology Research Center, Guangxi Medical University Cancer Hospital, Nanning, China

**Keywords:** immune checkpoint inhibitors (ICI), immune tolerance, hepatocellular carcinoma (HCC), tyrosine kinase inhibitors (TKIs), overall survival (OS), progression-free survival (PFS)

## Abstract

Hepatocellular carcinoma (HCC) is one of the most common malignant tumor in the world and its incidence is increasing in many countries. In recent years, with the deepening understanding of the immune and pathological mechanisms of HCC, immunotherapy based on the regulation of tumor immune microenvironment has become a new treatment choice for patients with HCC. Immune checkpoint inhibitors (ICIs) targeting programmed death protein-1, programmed death protein-ligand-1, or cytotoxic T-lymphocyte-associated antigen 4 are the most widely used. Instead of general immune-enhancing therapies, ICIs can reactivate anti-tumor immune responses by disrupting co-inhibitory T cell signaling. In this review, the research progress and existing problems of ICIs in the treatment of HCC in recent years are reviewed.

## Introduction

Hepatocellular carcinoma (HCC) is one of the most common malignant tumors in the world (1). Chronic hepatitis B (HBV) or C virus (HCV) infection is the main risk factors for the occurrence of HCC. Currently, the main curative treatments for HCC include surgical resection, liver transplantation and radiofrequency ablation (RFA) (2). However, due to the occult incidence of HCC, most patients have encountered with intermediate or advanced stage disease at the time of diagnosis and missing the best time for radical treatment (3). Lack of safe and effective treatments for intermediate or advanced stage HCC lead to rapid disease development and increasing mortality rate of patients (4).

Liver is not only an important immune regulatory organ of human body, but it is also a special immune tolerance organ (5). Immune tolerance is necessary for the liver to overcome the autoimmune protection mechanism of intestinal antigen overstimulation. However, its immunosuppressive microenvironment reduces the immune response rate of tumor cells and promotes the immune escape of tumor (6). In recent years, with the deepening understanding of the immune microenvironment of liver tumors, immunotherapy using the immune mechanism of the body to enhance tumor immune response and block tumor immunosuppression has become a new direction for the treatment of HCC, among which the immune checkpoint inhibitors (ICIs) have been most widely used (7, 8). ICIs reactivate the anti-tumor immune response by disrupting co-inhibitory T cell signaling (9, 10). This review will discuss the research progress and existing problems of ICIs monotherapy or combination therapy in the treatment of HCC in recent years.

## Immune Regulation of Liver and the Mechanism of HCC Occurrence and Development

**Figure 1:**
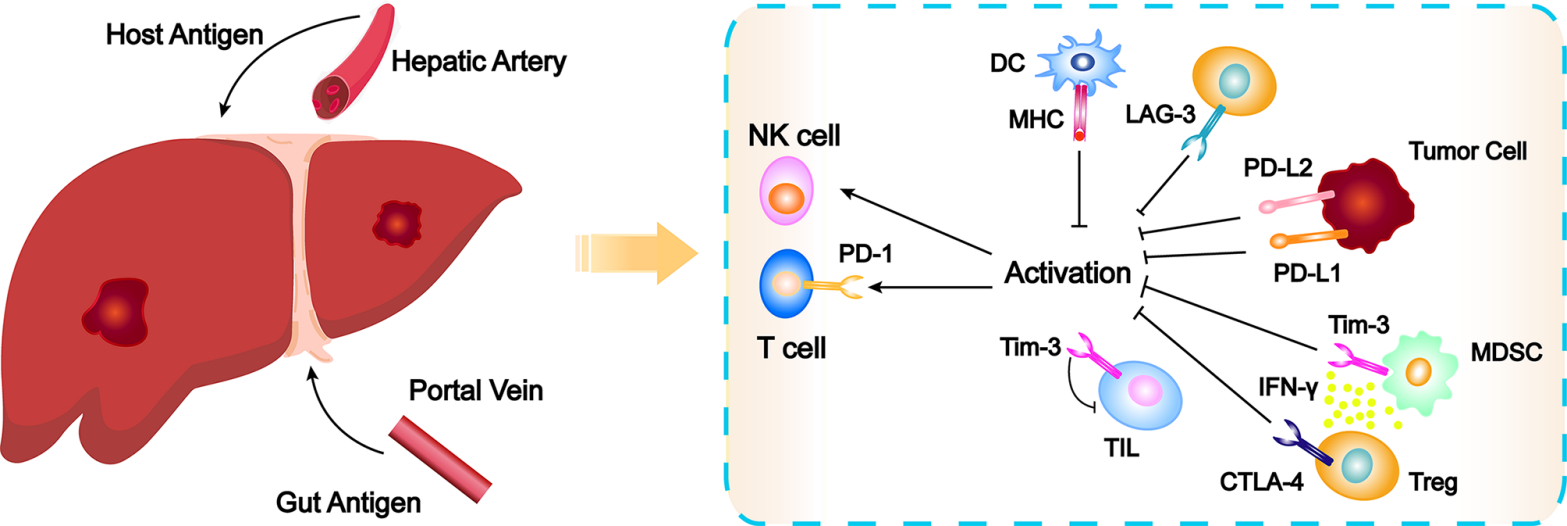
Schematic overview of the elements leading to immune exhaustion and checkpoint inhibitor action mechanisms. CTLA-4, cytotoxic T-lymphocyte-associated antigen 4; DC, dendritic cell; IFNγ, Interferon γ; LAG-3, Lymphocyte-activation gene 3; MDSC, myeloid-derived suppressor cell; MHC, major histocompatibility complex; NK, natural killer cell; PD-1, programmed cell death protein 1; PD-L1, programmed death protein-ligand-1; PD-L2, programmed death protein-ligand-2; TIL, tumor infiltrating lymphocytes; Tim-3, T cell immunoglobulin-3; Treg, regulatory T cell.

The liver is supplied by the hepatic artery and the portal vein. To avoid the endogenous antigens and the digestive tract of pathogenic microorganisms and their metabolic products to the liver through hepatic artery and portal vein system stimulate the immune system caused by excessive immune response, the microenvironment of liver through a variety of ways to establish immune tolerance, which maintain the steady state between inflammation and immune, avoid excessive immune reaction leads to liver cell damage (**Figure 1**) (11, 12). It is generally believed that chronic infection is the initial factor that causes sustained liver injury and then gradually develops into HCC through chronic hepatitis and cirrhosis (13). The interaction between inflammatory cells and immune cells makes the immune microenvironment more complex. The unique immune tolerance mechanism of liver leads to the immune escape of tumor, thus promoting the occurrence and development of HCC (14, 15). Under normal circumstances, the body mainly recognizes and kills tumor cells through cellular immunity dominated by T cells (16). Tumor associated antigens are released during the growth of tumor cells, which are presented by major histocompatibility complex I/II (MHC I/II) and recognized by antigen presenting cells (APCs). Then CD8^+^ T cells can be produced to exert cytotoxic effect on tumor cells. However, MCH I/II in HCC often functionally depleted, unable to induce the activation of CD8^+^ T cells, which leads to tumor immune escape (17). In addition, complete T cell activation requires the co-stimulation of the B7 molecule on APCs and the CD28 molecule receptor on T cells, while HCC downregulates the expression of co-stimulatory molecular receptors such as B7.1/B7.2, leading to the escape of tumor immunity (18). There are also a large number of immunosuppressive cells such as myeloid-derived suppressor cells (MDSCs) and regulatory T cells (Tregs) in the tumor microenvironment of HCC, which directly inhibit the tumor killing effect of natural killer cell (NK) and CD8^+^ T cells through overexpression of multiple factors (19). At the same time, in order to prevent excessive immune response from injuring normal hepatocytes, there are some immunoregulatory proteins on the surface of many immune cells, such as programmed cell death protein 1 (PD-1), which can transmit inhibitory downstream signals after binding with programmed death protein-ligand-1 (PD-L1) expressed on HCC cells or programmed death protein-ligand-2 (PD-L2) expressed on immune cells, resulting in a immunosuppression and immune tolerance environment. Thereby, tumor cells can escape the killing effect of T cells. Conversely, PD-L1 formed on the surface of HCC cells can not only bind to PD-1, but also bind to B7.1 on dendritic cells (DCs) to prevent the interaction of B7.1/CD28 to inhibit the activation of anti-tumor T cells, thereby evading T encirclement and suppression of cells (20, 21). Farther, there is another immune checkpoint molecule cytotoxic T-lymphocyte-associated antigen 4 (CTLA-4) on Tregs, which can bind with CD80 and CD86 on APCs to inhibit the activation of T lymphocytes, leading to immune escape of HCC cells (22). Because of this unique immune tolerance mechanism of liver that forms a highly immunosuppressive microenvironment, the efficacy of traditional drugs is limited (23). Relevant preclinical studies have shown that the immune response of T cells can be improved by inhibiting PD-1/PD-L1 or CTLA-4 (24, 25). Other studies found that application of ICIs treatment can enhance the killing effect on tumor cells (26, 27). The development of these studies has gradually revealed the complex immunosuppressive mechanism of HCC, which has laid a foundation for the clinical application of immunotherapy.

## ICIs Monotherapy in HCC

**Figure 2:**
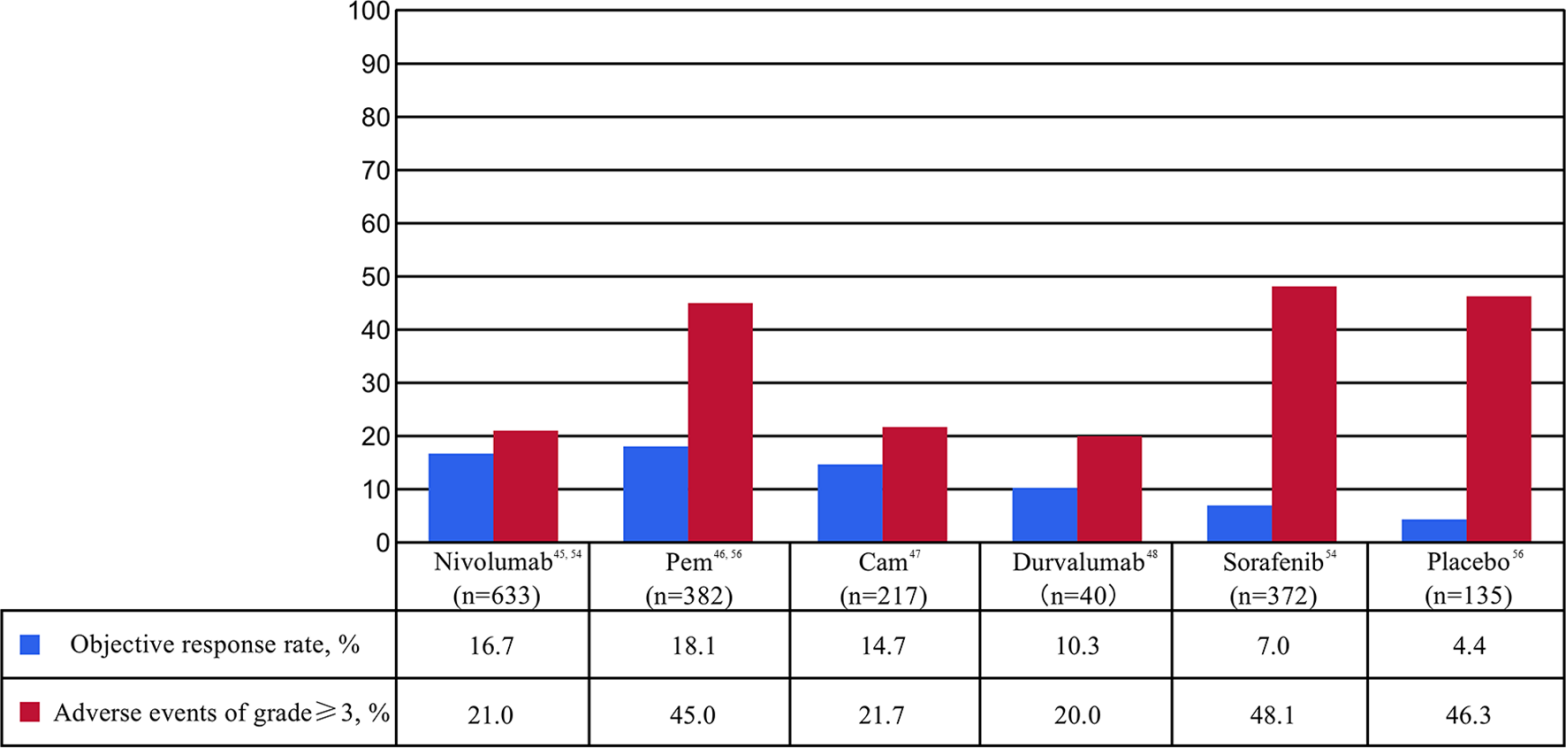
Percentages of objective response rate (ORR) and adverse events (AE) of at least grade 3 in clinical trials of immune checkpoint inhibitor monotherapy as first- or second-line for patients with advanced or unresectable hepatocellular carcinoma. Cam, camrelizumab; Pem, pembrolizumab.

ICIs are a series of molecules that are expressed on immune cells and regulate the degree of immune activation. They are essential for maintaining autotolerance, preventing autoimmune response and minimizing tissue damage by controlling the strength of immune response (28). Abnormal expression and function of immune checkpoint molecules are one of the important reasons for the occurrence of many cancers such as HCC (29, 30). For example, the overexpression or function of immune checkpoint molecules can inhibit the body immune function and promote the growth of tumor cells. On the contrary, when the inhibition function of the immune checkpoint molecules decreased, the self-organization might be damaged by the disturbance of the immune system (31, 32). ICIs are inhibitory drugs developed for immune checkpoint. ICIs can restore or enhance the killing effect of immune cells on tumor cells by blocking the inhibition of tumor cells expressing immune checkpoint on immune cells and achieve the purpose of tumor treatment (33). The following is a detailed description of the major ICIs. The main findings of clinical trials of ICIs mAb in patients with HCC are shown in **Table 1** and **Figure 2**.

**Table 1:** Clinical trials of immune checkpoint inhibitor monotherapy as first- or second-line for patients with advanced or unresectable hepatocellular carcinoma.

Drug	Trials	Phase	Design	Follow-up duration (months)	ORR according to RECIST 1.1(%)	Median survival time (months)	Median PFS time(months)	AE of grade ≥3 (%)
**First-line**								
Nivolumab	CheckMate 040 (34)	I/II	Nivolumab dose-expansion 0.1–10mg/kg iv q2w (n=214) *vs* Nivolumab dose-escalation 3 mg/kg iv q2w (n=48)	NR	19.6 *vs* 14.6	Not reached *vs* 15.0	4.0 *vs* 3.4	18.7 *vs* 25.0
Nivolumab	CheckMate 459 (35)	III	Nivolumab 240 mg iv q2w (n=371) *vs* Sorafenib 400 mg oral bid (n=372)	22.8	15.4 *vs* 7.0	16.4 *vs* 14.7	3.7 *vs* 3.8	21.8 *vs* 48.1
**Second-line**								
Pembrolizumab	KEYNOTE 224 (36)	II	Pembrolizumab 200 mg iv q3w (n=104)	12.3	17.3	12.9	4.9	24.0
Pembrolizumab	KEYNOTE 240 (37)	III	Pembrolizumab 200 mg iv q3w (n=278) *vs* Placebo 200 mg iv q3w (n=135)	13.8 *vs* 10.6	18.3 *vs* 4.4	13.9 *vs* 10.6	3.0 *vs* 2.8	52.7 *vs* 46.3
Camrelizumab	NCT02989922 (38)	II	Camrelizumab 3 mg/kg oral q2w (n=109) *vs* Camrelizumab 3 mg/kg oral q3w (n=108)	12.5	11.9 *vs* 17.6	14.2 *vs* 13.2	2.3 *vs* 2.0	22.0
Durvalumab	NCT01693562 (39)	I/II	Durvalumab10 mg/kg oral q2w (n=40)	6.0	10.3	13.2	NR	20.0

AE, adverse events; bid, every two days; iv, intravenous; NR, not reported; ORR, objective response rate; PFS, progression-free survival; qd, every day; q2w, every 2 weeks; q3w, every 3 weeks.

### CTLA-4

CTLA-4 is the first ICI found to inhibit the immune response, mainly expressed on activated CD4^+^ T cells, CD8^+^ T cells, Tregs, and immature T cells. It is homologous to the T cell costimulatory protein CD28 and can directly transmit the inhibitory signal by binding with B7 or competitively bind CD80/CD86 on the surface of APCs with a greater affinity than CD28 to inhibit the function of T cells, so as to promoting the occurrence and development of tumors (40). Therefore, blocking CTLA-4 can counteract the above-mentioned immunosuppressive mechanism in the process of T cell activation. Exert anti-tumor effects through reactivate and enhance the immune efficacy of T cells against tumor cells (41).

Ipilimumab is a humanized IgG1 monoclonal antibody (mAb) against CTLA-4 receptor. In the study by Hodi and coworkers (42), patients with stage III/IV melanoma were treated with ipilimumab or glycoprotein 100 (gp100) respectively. The results showed that ipilimumab significantly improved the postoperative survival of patients with melanoma. Therefore, ipilimumab was approved by the US Food and Drug Administration (FDA) in 2011 for the treatment of advanced melanoma, and it was also the first ICI for cancer treatment. Tremelimumab, another humanized IgG2 mAb against the CTLA-4 receptor, provided reliable evidence for the treatment of HCC with ICIs in a 2013 single-arm phase II trial (43). A total of 20 patients with HCV-related HCC were enrolled. Among them, 42.9% were Child-Pugh B liver function and 23.8% had failed sorafenib treatment. The small trial showed that the partial response (PR) of tremelimumab was 17.6%, the disease control rate (DCR) was 76.4%, the median overall survival time (mOS) was 8.2 months, and the time to progression (TTP) was 6.5 months. Simultaneously, significant reduction in viral load was also observed. Although 45.0% of patients experienced an elevation of grade 3/4 alanine aminotransferase (ALT), most were transient and mild. Therefore, tremelimumab is regarded as a safe and well tolerated drug, and plays a key role in HCC progression control and antivirus (43). However, with the in-depth study on the mechanism of action of CTLA-4 antibody drugs, some studies found that the mechanism of action of CTLA-4 antibody drugs is not immune checkpoint hypothesis, but the therapeutic effect is achieved by targeting the clearance of Treg in tumors (44, 45). The objective response rate (ORR) of patients with CTLA-4 mAb monotherapy was low (43), so the combination of CTLA-4 mAb with other therapeutic methods may be the future direction of CTLA-4 antibody development in HCC (46, 47).

### PD-1/PD-L1

PD-1 is a membrane protein located on the surface of most immune cells and a member of the CD28 immunoglobulin superfamily. PD-1 mainly expresses on activating T cells, NK cells and DCs, which can negatively regulate the immune response and maintain the body’s own tolerance. PD-1 has two ligands, PD-L1 and PD-L2. PD-L1 is expressed on a variety of cells. When PD-1 on the surface of T cells binds to PD-L1 on the surface of tumor, it can block the costimulatory signal of TCR and CD28 receptor, inhibit T cells proliferation and cytokine secretion, and cause T cells failure. At the same time, it also can promote the differentiation and proliferation of Treg and finally lead to tumor immune escape (48). The expression levels of PD-1 and PD-L1 in tumors were significantly correlated with poor prognoses of patients with HCC (49, 50). Subsequently, a number of clinical trials on ICIs monotherapy for advanced HCC have found that PD-1/PD-L1 mAb was well tolerated, and the ORR in advanced HCC could reach to 10.0%-20.0% (34, 36, 38, 39). Therefore, PD-1/PD-L1 inhibitors such as nivolumab and pembrolizumab are recommended as second-line treatment for advanced HCC according to the HCC official guidelines (51–53).

Nivolumab is a humanized IgG4 mAb against the PD-1 receptor. The first trial to provide evidence for the treatment of advanced HCC with a PD-1 inhibitor was CheckMate 040 (34), an international multicenter, single-arm, multi-cohort dose escalation and expansion study. In this study, nivolumab was evaluated as a first-line treatment in advanced HCC patients with no previous sorafenib treatment or sorafenib intolerance and as a second-line treatment in patients who had disease progression after receiving sorafenib. A total of 262 patients with advanced HCC with or without HBV/HCV infection were enrolled. During the dose-escalation phase, the ORR, DCR, and median duration of response (mDOR) were 14.6%, 58.3%, and 17.0 months, respectively. In the dose-expansion phase (54) results showed that the ORR of first-line and second-line treatment with nivolumab was 22.5% and 16.2-19.3%, respectively, with a 12-month OS of 73.3% and 58.0-60.0%, respectively. Overall, responses occurred in patients regardless of etiology or tumor cell PD-L1 expression. The incidence of grade 3/4 adverse events (AE) was 28.8% in sorafenib treated group and 17.7% in sorafenib untreated group. The results of this study show the potential efficacy of nivolumab mAb in the treatment of advanced HCC. Nivolumab has been unanimously recommended by international guidelines (51–53) as the second-line treatment in advanced HCC based on these results, and it is the first ICI to be approved for the treatment of advanced HCC. Moreover, the efficacy and safety of CheckMate 040 in Asian patients were similar to that of the general population (55). CheckMate 459 (35, 56) is an international multicenter, randomized controlled phase III clinical trial, which aims to compare the clinical efficacy and safety of nivolumab *versus* sorafenib as the first-line treatment for patients with advanced HCC. The mOS in the novilumab and sorafenib groups were 16.4 and 14.8 months, respectively ([hazard ratio, HR] 0.85, 95%CI 0.72-1.00, *P*=0.052). And ORR was 15.4% in the novilumab group and 7.0% in the sorafenib group. The incidence of grade 3/4 AE in the novilumab and sorafenib groups was 22.3% and 49.6%, respectively. The primary endpoint of OS was not statistically significant may be related to the fact that 45.7% of patients in the sorafenib group received subsequent immunotherapy in the study. This study further suggests that novilumab is more effective and safer than sorafenib in the treatment of advanced HCC.

Pembrolizumab is another humanized IgG4 mAb against the PD-1 receptor and was the first PD-1 inhibitor to be approved for clinical use. KYENOTE-224 (36) is a non-randomized, international multicenter, open phase II clinical trial evaluating the efficacy and safety of pembrolizumab in patients with advanced HCC who have failed sorafenib. A total of 104 patients were enrolled in the trial, of which 21.2% had HBV infection and 25.0% had HCV infection. The results showed that mOS of pembrolizumab as second-line treatment was 12.9 months, median progression-free survival (mPFS) was 4.9 months, ORR was 17.3%, and DCR was 61.5%. In the subgroup analysis, ORR was similar in patients with or without HBV/HCV infection. In terms of safety, the incidence of AE of at least grade 3 was 24.0%, mainly manifested by elevated AST, ALT, fatigue, etc. Most of these AE were tolerable. In November 2018, the FDA accelerated approval of pembrolizumab as second-line treatment in advanced HCC. After that, KEYNOTE-240 (37), a phase III clinical trial of pembrolizumab as second-line treatment for advanced HCC, found pembrolizumab treatment can significantly improve OS and PFS, although the difference does not reach the preset statistical level. The mOS was 13.9 and 10.6 months (*P*=0.024), and the mPFS was 3.0 and 2.8 months (*P*=0.019), respectively. Meanwhile, the ORR of pembrolizumab group (18.3%) was significantly higher than that of placebo group (4.4%) (*P*<0.001). The safety is basically consistent with the results of KEYNOTE-224. Subgroup analysis showed that pembrolizumab significantly prolonged OS (13.8 vs 8.3 months, *P*<0.001) and PFS (2.8 vs 1.4 months, *P*<0.001) compared with placebo in Asian populations with more HBV infection and worse tumor stage. This further supports the status of pembrolizumab as a second-line treatment in advanced HCC. Currently, another phase III trial, KEYNOTE-394 (NCT03062358), is underway to evaluate the efficacy and safety of pembrolizumab as a second-line treatment for advanced HCC in Asian patients. The results of this trial are expected for the prevalence of HBV-associated HCC in Asia.

Camrelizumab is a humanized IgG4 mAb against PD-1 receptor independently developed in China. In 2016, a prospective, multicenter, randomized parallel controlled phase II clinical trial (38) was conducted in China to evaluate the clinical efficacy and safety of camrelizumab in the treatment of advanced HCC. A total of 220 advanced HCC patients were enrolled in this trial. Among them, the HBV infection rate was 83.4%. In addition, 81.6% of the patients had extrahepatic metastasis, 22.6% had received two or more lines of previous systemic treatments, 94.9% encountered with Barcelona Clinical Liver Cancer (BCLC) stage C, and 51.2% were with alpha fetoprotein ≥ 400 ng/mL. The overall ORR, DCR, mPFS, and mOS were 14.7%, 44.7%, 2.1 months, and 13.8 months. In terms of safety, the incidence of grade 3/4 AE was 21.7%. The most common immune-related AE was reactive cutaneous capillary endothelial proliferation (RCCEP), but most of them were grade 1-2 and mildly reversible. Further study found that the occurrence of RCCEP and curative effect has a strong correlation (57). In this trial, camrelizumab achieved similar efficacy with nivolumab and pembrolizumab in patients with worse baseline status. Based on this research, camrelizumab was officially approved by the National Medical Products Administration (NMPA) of China in March 2020 as a second-line treatment for patients with advanced HCC.

Durvalumab is a humanized IgG1 mAb with high affinity targeting PD-L1 that selectively blocks the binding of PD-L1 to PD-1 and CD-80/B7-1, thus enabling T cells to recognize and kill tumor cells. In a phase I trial of durvalumab (58), 408 patients with solid tumors, including 19 patients with advanced HCC, were enrolled. Durvalumab treated patients had a 21.0% DCR and 34.0% AE incidence (grade 3 AE, 5.6%; grade 4/5 AE, 0%). The favorable results of this study led to another phase I/II clinical trial (39), which included 40 patients with advanced HCC and 92.5% of whom had received sorafenib. The ORR, DCR, and mOS were 10.3%, 33.3%, and 13.2 months, respectively. Although 80.0% of patients experienced AE, the incidence of grade 3/4 AE was only 20.0%. This trial further supports the development prospect of durvalumab mAb for advanced HCC. At present, atezolizumab (59), avelumab (60) and other monoclonal antibodies targeting PD-L1 receptors for the treatment of HCC are undergoing.

## ICIs Combination in HCC

**Figure 3:**
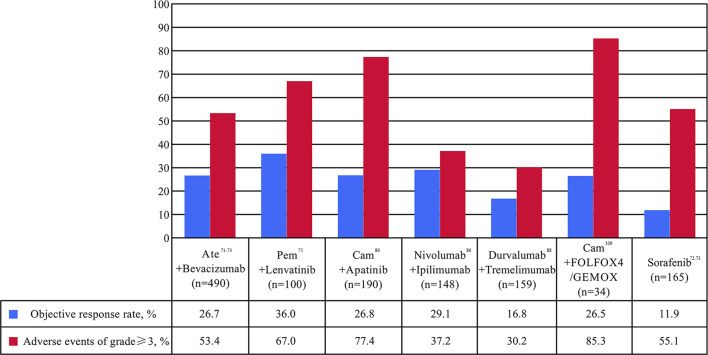
Percentages of objective response rate (ORR) and adverse events (AE) of at least grade 3 in clinical trials of immune checkpoint inhibitor combination as first- or second-line for patients with advanced or unresectable hepatocellular carcinoma. Ate, atezolizumab; Cam, camrelizumab; FOLFOX4, intravenously infusional fluorouracil, leucovorin, and oxaliplatin; Pem, pembrolizumab.

With the continuous understanding of the tumor microenvironment of HCC, immunotherapy, especially ICIs, has become a new method for the effective treatment of HCC. However, the local tumor immune tolerance microenvironment hinders the performance of immunotherapy, leading to the low efficacy of ICIs monotherapy, with an ORR for only 10.0-20.0%, and the remission time of some patients is limited (61, 62). In particular, the recent failure of phase III clinical trials of ICIs monotherapy impact on first-line (35) and second-line (37) treatment of HCC indicate that it may be necessary to combine other drugs to improve the efficacy. A series of studies have shown that ICIs combined with immunotherapy with different mechanisms can help ameliorate the tumor immune microenvironment, and then improve patient response rate and anti-tumor effect, which may be the future development focus of HCC immunotherapy (63–65). The following is a detailed introduction of ICIs combination therapy. The main clinical trial results of ICIs combination therapy in patients with HCC are shown in **Table 2** and **Figure 3**.

**Table 2:** Clinical trials of immune checkpoint inhibitor combination as first- or second-line for patients with advanced or unresectable hepatocellular carcinoma.

Drug	Trials	Phase	Design	Follow-up duration (months)	ORR according to RECIST 1.1 (%)	Median survival time(months)	Median PFS time(months)	AE of grade ≥3 (%)
**First-line**								
Pembrolizumab + Lenvatinib	KEYNOTE 524 (66)	Ib	Pembrolizumab 200 mg iv q3w + Lenvatinib 12 mg (for bodyweight≥60 kg) or 8 mg (for bodyweight<60 kg) oral qd (n=100)	10.6	36.0	22.0	9.3	67.0
Atezolizumab + Bevacizumab	GO30140 (67)	Ib	Atezolizumab 1200 mg iv + Bevacizumab 15 mg/kg iv q3w (n=104) *vs* Atezolizumab 1200 mg iv + Bevacizumab 15 mg/kg iv q3w (n=60) *vs* Atezolizumab 1200 mg iv q3w (n=59)	12.4 *vs* 6.6 *vs* 6.7	35.6 *vs* 20.0 *vs* 16.9	17.1 *vs* not reached *vs* not reached	7.3 *vs* 5.6 *vs* 3.4	52.9 *vs* 36.7 *vs* 13.6
Atezolizumab + Bevacizumab	IMbrave 150 (68, 69)	III	Atezolizumab 1200 mg iv + Bevacizumab 15 mg/kg iv q3w (n=336) *vs* Sorafenib 400 mg oral bid (n=165)	8.6 *vs* 8.1	27.3 *vs* 11.9	Not reached *vs* 13.2	6.8 *vs* 4.3	56.5 *vs* 55.1
Camrelizumab + Apatinib	RESCUE (70)	II	Camrelizumab 200 mg (for bodyweight≥50 kg) or 3 mg/kg (for bodyweight<50 kg) iv q2w + Apatinib oral 250 mg qd (n=70)	16.7	34.3	Not reached	5.7	78.6
**Second-line**								
Nivolumab+ Ipilimumab	CheckMate 040 (71)	I/II	Nivolumab1 mg/kg iv+ Ipilimumab 3mg/kg iv q3w (4 doses) (n=50) *vs* Nivolumab 3 mg/kg iv + Ipilimumab 1mg/kg iv q3w (4 doses), each followed by Nivolumab 240 mg iv q2w (n=49) *vs* Nivolumab 3 mg/kg iv q2w + Ipilimumab 1 mg/kg iv q6w (n=49)	30.7	32.0 *vs* 26.5 *vs* 28.6	22.8 *vs* 12.5 *vs* 12.7	–	53.1 *vs* 28.6 *vs* 31.3
Camrelizumab + Apatinib	RESCUE (70)	II	Camrelizumab 200 mg (for bodyweight≥50 kg) or 3 mg/kg (for bodyweight<50 kg) iv q2w + Apatinib oral 250 mg qd (n=120)	14.0	22.5	Not reached	5.5	76.7
Durvalumab + Tremelimumab	Study 22 (72)	II	T 300 mg oral+ D 1500 mg oral 1 dose followed by D 1500 mg oral q4w (n=75) *vs* T 75 mg oral q4w + D 1500 mg oral q4w (4 doses) followed by D 1500 mg oral q4w (n=84) *vs* D 1500 mg oral q4w (n=104) *vs* T 750 mg oral q4w (n=69)	11.7 *vs* 14.6 *vs* 8.9 *vs* 15.8	22.7 *vs* 9.5 *vs* 9.6 *vs* 7.2	18.7 *vs* 11.3 *vs* 11.7 *vs* 17.1	NR	35.1 *vs* 24.4 *vs* 17.8 *vs* 42.0
Camrelizumab + FOLFOX4/GEMOX	NCT03092895 (73)	II	Camrelizumab 3 mg/kg iv q2w + FOLFOX4/GEMOX (n=34)	NR	26.5	Not reached	5.5	85.3

AE, adverse events; bid, every two days; iv, intravenous; NR, not reported; ORR, objective response rate; PFS, progression-free survival; qd, every day; q2w, every 2 weeks; q3w, every 3 weeks.

### ICIs Combined With Antiangiogenic Drugs

Antiangiogenic therapy is another tumor treatment method. Currently, the antiangiogenic drugs used in clinic mainly include vascular endothelial growth factor (VEGF), its receptor VEGFR, and VEGFR tyrosine kinase inhibitors (TKIs), etc. (74) As a vascular rich tumor, the special structure of the new vessel wall of HCC often makes it difficult for anti-tumor drugs and immune cells to reach the tumor site. Anti-angiogenic drugs can normalize immunosuppressed tumor blood vessels by targeting the antagonistic VEGF and VEGFR pathways (75). They can also reactivate antigen-presenting DCs, promote the activation, infiltration and migration of lymphocytes and reduce the recruitment of inhibitory cells such as Tregs and MDSCs, finally, avoids the depletion of effector T lymphocytes (76). Combination therapy with anti-angiogenic drugs and ICIs will improve the sensitivity of tumor to angiogenesis drugs, create favorable environment for ICIs treatment and play a synergistic role in treatment (77). Therefore, it is of great significance to further explore the efficacy of antiangiogenic drugs combined with ICIs in HCC.

#### Atezolizumab Combined With Bevacizumab

Atezolizumab is the first PD-L1 inhibitor approved for marketing targeting the PD-L1 receptor (59), while bevacizumab is a humanized IgG1 mAb against VEGF (78). The combined treatment of VEGF and PD-L1 can block the VEGF and PD-L1 pathways at the same time and produce synergistic anti-tumor effects. A phase Ib GO30140 (67) trial of atezolizumab alone (n=59) *versus* atezolizumab combined with bevacizumab (n=164) in patients with advanced HCC found that atezolizumab combined with bevacizumab group had the best effect. The primary endpoint of ORR has reached 35.6%. The DCR, mOS, and mPFS was 71.2%, 17.1 months, and 7.3 months, respectively. Moreover, atezolizumab combined with bevacizumab was well tolerated and had manageable toxicity, with grade 3/4 AE accounting for 52.9%. No new safety problems were found except for the known safety events of atezolizumab and bevacizumab. Based on the above results, the FDA has awarded atezolizumab combined with bevacizumab as a breakthrough drug for the first-line treatment for patients with advanced or metastatic HCC. In addition, a multicenter, randomized controlled phase III clinical trial IMbrave150 (68, 69) based on this study further supports the application of “T+A” regimen in advanced HCC. The atezolizumab-bevacizumab group had significantly better OS and PFS rate than sorafenib group (all *P*<0.001), mOS of the two groups was 19.2 and 13.4 months, respectively (HR 0.66, 95%CI 0.52 to 0.85, *P*<0.001). The corresponding mPFS was 6.8 and 4.3 months, respectively (HR 0.59, 95%CI 0.47 to 0.76, *P*<0.001); ORR was 27.3% and 11.9%, respectively; DCR was 73.6% and 55.3%, respectively. In terms of safety, the incidence of AE in “T+A” regimen was basically the same as that of sorafenib treatment, with 56.5% and 55.1% in AE above grade 3, respectively. The IMbrave150 trial (68, 69) makes atezolizumab combined with bevacizumab a new standard first-line treatment for advanced HCC recommended by several international HCC guidelines (51–53), and also opens the door of ICIs combined with antiangiogenic drugs in the treatment of HCC.

#### ICIs Combined With Lenvatinib

Lenvatinib is a TKIs that inhibits the growth of tumor neovascularization and achieves anti-cancer effect by antagonizing VEGFR1-3, FGFR1-4, PDFGR-α, RET and KIT targets. Lenvatinib has been approved as a first-line treatment for advanced HCC based on the REFLECT trial (79). KEYNOTE-524 (66), a phase Ib clinical trial evaluating the safety and efficacy of pembrolizumab in combination with lenvatinib in the treatment of unresectable HCC. The first part of this study (n=100) showed that ORR was 36.7%. Seven (7.0%) patients discontinued treatment due to AE and no new safety signals were identified. At the second part of the study, the included population has expanded to 104, of which 100 were included in the analysis. The results showed that the mOS, mPFS, ORR, and DCR was 22.0 months, 8.6 months, 36.0%, and 88.0%, resepectively. The incidence of AE above grade 3 was 67.0%. Among them, 3 patients died because of treatment-related factors, but the overall situation was controllable and manageable. Based on these results, the FDA granted breakthrough drug status to pembrolizumab in combination with lenvatinib for the first-line treatment of advanced unresectable HCC in July 2019. A recent retrospective study confirmed the safety and efficacy of ICIs combined with lenvatinib in patients with unresectable HCC (80). This study included 65 patients with ICIs combined with lenvatinib therapy and 45 with lenvatinib monotherapy. ICIs plus lenvatinib provided significantly higher OS (HR 0.47, 95%CI 0.26 to 0.85, *P*=0.013) and PFS (HR 0.35, 95%CI 0.20 to 0.63, *P*<0.001) than lenvatinib monotherapy. Moreover, patients with combination therapy had significantly higher ORR (41.5% vs 20.0%, *P*=0.023) and DCR (72.3% *vs* 46.7%, *P*=0.009) than those with lenvatinib. No treatment-related deaths were observed. Grade 3 or greater AE in either treatment group were similar. Therefore, ICIs plus lenvatinib in real-world study showed significantly promising efficacy and manageable safety than lenvatinib alone in patients with unresectable HCC. Nowadays, LEAP-002 (81), an international multicenter, randomized, double-blind phase III trial aimed at exploring the efficacy and safety of pembrolizumab combined with lenvatinib in the first-line treatment of advanced HCC is being carried out. It may provide additional options for patients with HCC.

#### Camrelizumab Combined With Apatinib

Apatinib is another type of TKIs that inhibits the formation of tyrosine kinase and inhibits the neoangiogenesis of tumor tissue through highly selective competition for ATP binding sites of VEGFR-2 in cells, so as to achieve the purpose of anti-tumor. Based on the AHELP study (82), apatinib has been approved by the NMPA of China for second-line treatment in advanced HCC. A phase I clinical trial (n=18) (83) first evaluated the effect of camrelizumab combined with apatinib in the treatment of HCC. The results showed that ORR was 50.0%, DCR was 93.8% and the mPFs was 5.8 months. The mOS has not reached. The main grade 3/4 AE was hypertension. At the same time, a non-randomized, open, multi-center phase II clinical trial RESCUE (70) was launched to evaluate the effectiveness and safety of combination therapies in the first-line and second-line treatment of HCC. The results showed that the ORR of the first-line and the second-line treatment group was 34.3% and 22.5%, respectively. The mPFS of the two groups were 5.7 and 5.5 months, and the corresponding 12-month OS was 74.7% and 68.2%, respectively. The incidence of grade 3 and above AE was 78.6% in the first-line treatment group and 76.7% in the second-line treatment group, which was consistent with previous reports. In addition, findings from retrospective study (84) confirmed that camrelizumab combined with apatinib yielded a promising outcome in patients with HCC involving portal vein tumor thrombus. Currently, an international multi-center, randomized controlled III clinical trial (NCT03764293) of camrelizumab combined with apatinib *versus* sorafenib in first-line treatment of advanced HCC is in progress. However, the above studies was conducted primarily in patients with CHB, which may limit the application of the findings to other patient populations. Trials recruting patients with HCC and other background liver disease, such as HCV or alcoholic cirrhosis, are needed.

### ICIs Combination Therapy

Although ICIs have been widely used in advanced HCC, PD-1/PD-L1 mAb fail to perform its intended function due to the lack of activated CD8^+^ T cells in some patients. Different ICIs have different mechanisms of action in immune pathways. For example, PD-1/PD-L1 mAb combined application of CTLA-4 inhibitors can induce CD8^+^ T cells proliferate and reactivate to kill tumor cells (85). James Allison, the discoverer of CTLA-4 and the 2018 Nobel laureate, has published a paper in *Cell* supporting the mechanism of action of ICIs combined therapy for HCC in 2017 (86). Preclinical studies on solid tumors based on the above theories have shown that the combination therapy has higher response rate and better curative effect compared with monotherapy (87, 88). As dual-immunotherapy was approved by FDA in 2011 and 2017 for malignant melanoma and renal cell carcinoma respectively, it also indicates that dual-immunotherapy has become an important model to improve the clinical efficacy of ICIs in malignant tumors.

#### Nivolumab Combined With Ipilimumab

CheckMate 040 (71) trial evaluated the safety and efficacy of nivolumab combined with ipilimumab in sorafenib treated patients with advanced HCC. A total of 148 patients with advanced HCC were included in this study, of whom 33.8% had macrovascular invasion, 82.4% had extrahepatic spread, and 91.5% had BCLC stage C disease. Patients were randomly assigned to three different dose groups. The results showed that the ORR and DCR were 29.1% and 43.9% in the total population. Among them, group A (nivolumab 1 mg/kg+ipilimumab 3 mg/kg+ sequential nivolumab 240 mg maintenance therapy) had the best OS, with mOS of 22.8 months and 30-month OS rate of 44.0%. Safety analysis showed that grade 3/4 AE occurred in 37.0%. Among them, 5.0% were discontinued therapy. The most common AE were pruritus and rash. However, the safety of the combination regimen was controllable, and no new safety signals were observed in different dose groups. This trial confirmed for the first time that ICIs combination therapy is effective and well tolerated in advanced HCC. Based on the aforementioned results, the FDA approved nivolumab combined with ipilimumab for patients with advanced HCC who had previously treated with sorafenib in March 2020. CheckMate 9DW (NCT04039607), a phase II trial, is currently underway to evaluate the efficacy and safety of nivolumab in combination with ipilimumab in first-line treatment of patients with advanced HCC.

#### Durvalumab Combined With Tremelimumab

Durvalumab in combination with tremelimumab is another dual-immunotherapy regimen being explored in HCC. A phase I/II trial (89) enrolled 40 patients with advanced HCC (30% untreated with sorafenib) and explored the role of durvalumab combined with tremelimumab in such patients. The overall ORR was 15.0% and the 16-week DCR was 57.5%. The incidence of AE above grade 3 (60.0%) was increased compared with monotherapy (39), but the safety profile was deemed tolerable and no new AE events occurred. This trial initially shows that durvalumab combined with tremelimumab is promising for the treatment of patients with advanced HCC. Based on the results of this study, the team then carried out an international multicenter, open label, randomized controlled phase II clinical trial Study22 (72), which evaluated the safety and efficacy of tremelimumab and durvalumab as monotherapies and durvalumab combined with tremelimumab regimens in patients with advanced HCC. A total of 322 patients were enrolled and randomly divided into four groups. The results showed that clinical benefits were observed in all the treatment groups, among which tremelimumab 300 mg + durvalumab 1500 mg group had the most obvious advantages. The mOS was 18.7 months, ORR reached 22.7% and DOR was not achieved. The incidence of grade 3/4 AE was 35.1%. Based on the preliminary results of this trial, the FDA awarded durvalumab and tremelimumab orphan drugs for the treatment of HCC on January 20, 2020. And also based on this study, a HIMALAYA (90) Phase III trial is being conducted to explore the efficacy and safety of durvalumab combined with tremelimumab in the first-line treatment of advanced HCC.

### ICIs Combined With Other Treatments

In addition to the above-mentioned attempts of combination therapy, the combined application of immunotherapy with chemoradiotherapy, TACE, RFA and other related studies are becoming the focus in the field of HCC (91). Tumors with low mutation load and fewer neoantigens generally have lower immunogenicity and low or even no response to ICIs (92). Some basic studies (93–95) have found that radiotherapy, chemotherapy, TACE, RFA and other treatments can induce local inflammatory response, change the tumor immune microenvironment and reactivate the immune response of patients by exposing and releasing tumor antigens after killing tumor cells. Then the combination of ICIs can further maintain or enhance the function of antigen presenting cells to activate T cells, thereby enhancing the anti-tumor effect.

#### ICIs Combined With TACE/RFA

A phase I/II trial (96) evaluated tremelimumab in combination with TACE/RFA for the treatment of advanced HCC. Of the 19 evaluable patients, 26.3% were able to obtain PR. Significant reduction in viral load was found in 12 of the 14 patients with HCV. Tumor biopsies 6 weeks after treatment showed that CD8^+^ T in tumor lesions of patients with clinical benefit were clear increased. The 6-month and 12-month PFS rate was 57.1% and 33.1%, respectively. The mTTP was 7.4 months and the mOS was 12.3 months. The main AE was pruritus and no new AE were observed. This trial preliminarily confirmed the efficacy and safety of ICIs combined with TACE/RFA in the treatment of advanced HCC. In addition, a phase I clinical trial (97) to evaluate novilumab combined with drug-eluting bead transarterial chemoembolization (DEB-TACE) for the treatment of HCC patients included 9 patients with BCLC stage B and Child-Pugh grade A. The results showed that 2 of the 9 evaluable patients achieved PR and 2 cases were actually SD. The 6-month and 12-month PFS was 53.0% and 40.0%, respectively. And the 12-month OS was 71.0%. Grade 3 AE is mainly associated with elevated transaminase. At present, immunotherapy combined with locoregional therapies is still in the exploratory stage and a number of clinical studies with similar mechanisms have been carried out, including CheckMate 74W (46), EMERALD-1 (98), LEAP-002 (99), etc. Hoping these studies can fill the gap of immunotherapy combined with locoregional therapy in the treatment of HCC.

#### ICIs Combined With Radiotherapy

Due to the progress made in recent years, radiotherapy has been listed as one of the treatment methods for unresectable HCC patients by several international guidelines for HCC (51–53). Many studies (100, 101) have found that stereotactic body radiotherapy (SBRT), which stimulates immune response through the formation of antitumor antibodies and abscopal effect, is a safe and effective locoregional treatment for advanced HCC. As immunotherapy continues to heat up in the field of cancer, researches on ICIs combined with radiotherapy have been carried out. In other solid tumors, especially NSCLC, ICIs combined with radiotherapy has shown well synergistic effects (102). Kim et al. (103) prospectively investigated the relationship between the serum level of soluble PD-L1 in blood and prognosis in HCC patients receiving radiotherapy. High serum levels of soluble PD-L1 were found to correlate with tumor aggressiveness (tumor size and stage) and poor prognosis of HCC, while serum levels of soluble PD-L1 were significantly increased in HCC patients treated with SBRT (P<0.001). This study suggests that ICIs combined with radiotherapy may be a potential treatment option for HCC. Chiang et al. (104) found that after using nivolumab combined with radiotherapy in 5 patients with advanced HCC, the ORR reached 100%, including 40.0% CR and 60.0% PR. The median tumor diameter reduction rate was 38.7%, mPFS was 14.9 months, 1-year OS and 1-year local control rate were both 100%. Only 1 patient developed grade 3 AE, suggesting that this combination therapy has considerable efficacy and safety. In addition, a retrospective study (n=76) (105) found that the PFS (*P*=0.008) and OS (*P*=0.007) of HCC patients treated with the combination therapy were significantly higher than those treated with nivolumab alone, with a good safety profile. All the aforementioned studies have shown that ICIs combined with radiotherapy is an effective way to treat advanced HCC. Based on this, several other randomized controlled trials such as NCT03033446 and NCT02239900 are currently on-going.

#### ICIs Combined With Chemotherapy

HCC has high heterogeneity (106), which leads to traditional chemotherapy drugs can not benefit patients. However, Some studies (107, 108) have found that HCC can restart the tumor immune response after receiving chemotherapy, making it from “immune cold tumors “ to “immune hot tumors “. At this time, combined with ICIs may improve its curative effect. Qin s et al. (73) reported the results of a multicenter phase II clinical study (n=34) of camrelizumab combined with FOLFOX4/GEMOX system chemotherapy as a first-line treatment of advanced HCC. Among 34 evaluable HCC patients, 79.4% had HBV infection. The ORR and DCR were 26.5% and 79.4%, respectively. The mPFS reached 5.5 months and TTR was 2.0 months. The mOS and DOR had not yet reached. The incidence of AE above grade 3 was 85.3% and most commonly neutrophil count decreased, but the safety was controllable. The efficacy and safety of camrelizumab combined with FOLFOX4/GEMOX chemotherapy were demonstrated in this trial, which may provide a new treatment option for patients with advanced HCC. At present, a phase III clinical trial of camrelizumab combined with FOLFOX4 *versus* sorafenib/FOLFOX4 in the first-line treatment of patients with advanced HCC is ongoing.

## Challenges Faced by ICIs in HCC Application

### Problems in the Efficacy Evaluation of ICIs Clinical Trials

The high heterogeneity (106) and variability of the tumor microenvironment hinders the immune response mediated by ICIs, which makes the time and intensity of ICIs vary in HCC patients. At the same time, ICIs does not kill tumor cells directly, but plays an anti-tumor role by reversing the immunosuppressive state and reactivating the immune system response. Therefore, its clinical effect often needs a certain time to be reflected (109). Some patients abandoned ICIs therapy after their tumors grew larger in the first few months of treatment, thinking they are progressing. However, part of them may be pseudo-progression (110) (the phenomenon that the target lesions show an obvious growth trend or appear with new lesions in the imaging evaluation after the first anti-tumor treatment, but the target lesions remain stable, shrink or disappear in the subsequent evaluation control). Therefore, a long enough window of clinical observation is needed to evaluate the antitumor activity of ICIs in clinical trials, which will avoid the pseudoprogression of the continuous use of potentially effective therapeutic agents due to false progression and reduce the survival time and quality of life that patients originally enjoyed. Otherwise, clinical trial endpoint OS is affected by a variety of complex factors, such as follow-up time, follow-up treatment and so on, which may make OS as the main end point of clinical trials to evaluate the treatment effect may be easily misinterpreted and it needs sufficient follow-up time to evaluate the efficacy of ICIs. It is even possible that the failure of some clinical trials may be related to the follow-up treatment of the control group (111). For example, after the failure of sorafenib treatment or intolerance, the control group tended to follow the guidelines for sequential treatment with PD-1 preparations. Sequential therapy often prolongs OS of patients in the control group, leading to failure of the trial without a positive result (35, 37). This may require us to re-examine the ability of different endpoints in clinical trials to evaluate the outcome of patients.

### Complications of ICIs

Although current oncology immunotherapies targeting the immune checkpoint pathway have achieved high objective results, there are still some potential serious AE in ICIs (112). As ICIs combination regimens have been developed, toxic side effects have increased. Although they are usually controllable, they may still be life-threatening. Studies have shown that for PD-1/PD-L1 mAb, the overall incidence of AE was generally dose-independent, at 27.0% and AE above grade 3 was 6.0%. For CTLA-4 mAb, the overall incidence of AE varied with dose, at 72.0% and the incidence of AE above grade 3 was 24.0%. Among them, the skin, gastrointestinal, liver, lungs and endocrine systems are the most common (113). Skin toxicity is the earliest and most recurrent AE in ICIs, which is generally manifested as rash, pruritusand vitiligo, mostly on the limbs and trunk. The incidence of skin toxicity is approximately 30.0% in patients treated with PD-1/PD-L1 mAb and as high as 40.0% in patients treated with CTLA-4 mAb. Gastrointestinal toxicity is the second-most common side-effect of AE with diarrhea and colitis being the most prominent symptoms. Severe colitis may even lead to perforation of the colon and peritonitis, which can be life-threatening. The incidence of gastrointestinal toxicity is approximately 10.0-20.0% in patients receiving PD-1/PD-L1 mAb and 30.0% in patients receiving CTLA-4 mAb, respectively, while the incidence is approximately 30.0-40.0% when anti-CTLA-4 mAb are combined with PD-1/PD-L1 mAb. Hepatotoxicity is also one of the common AE in ICIs, mainly manifested as hepatitis, which may lead to liver failure and even death in severe cases. The incidence of hepatotoxicity was as high as 15.0% in patients receiving CTLA-4 mAb while 5.0-10.0% in patients receiving PD-1/PD-L1 mAb. PD-1/PD-L1 mAb typically cause the elevation of serum transaminase, while CTLA-4 mAb may cause an increase in alkaline phosphatase, gamma-glutamyltransferase, or bilirubin in patients with cholestasis (114). Cardiotoxicity has emerged as an infrequent but often lethal complication of HCC, mainly manifested as myocarditis and pericarditis (115). The incidence of cardiac complications is estimated to be 0.27% to 1.14% (116). Heinzerling et al. (117) found that patients who have suffered from cardiac pathological changes and peripheral artery diseases are more prone to cardiotoxic complications after treatment with ICIs even after their condition has been stable for many years. In addition, PD-1 knockout mice produce autoantibodies against cardiac myosin, resulting in fatal immune myocarditis in mice with autoimmune deficiency (118). Although patients with cardiac disease and potential autoimmune diseases were excluded from clinical trials due to strict restrictions on the inclusion criteria of clinical trials. However, cardiotoxicity has been reported after treatment with pembrolizumab (36). Otherwise, in a retrospective analysis (119), the combination of nivolumab and ipilimumab increased the risk of developing myocarditis by 4.74 times compared with nivolumab alone. Therefore, the real-world risk of cardiac AE in patients with cardiac disease and potential autoimmune diseases treated with ICIs is still unclear. We should pay more attention to this problem, early recognise and prompt intervene in it. At present, the treatment of AE in ICIs is mainly to stop medication and give symptomatic supportive treatment. Although the AE in ICIs are diverse and have different toxicity, most of them are reversible and controllable. We still need to focus on the occurrence of AE that may lead to serious consequences (120).

### Prediction of Efficacy of ICIs Treatment

With various studies and clinical trials underway, the potential of ICIs in the treatment of HCC has been widely recognized. However, data analysis found that not all patients receiving ICIs treatment could obtain lasting clinical efficacy. This suggests that we still need to work on finding biomarkers that can suggest a good response to ICIs (121). Harding et al. (122) found that Wnt/CTNNB1 mutations could indicate whether patients would benefit from PD-1/PD-L1 mAb therapy. In this study, the second-generation gene sequencing was performed on tumor samples from 127 patients with advanced HCC. Among the 27 patients who could be evaluated for ICIs treatment, it was found that all HCC patients with Wnt/CTNNB1 mutations were insensitive to PD-1/PD-L1 mAb, and mOSs and mPFs were lower than those without mutations. It is suggested that Wnt/CTNNB1 mutation is related to ICIs resistance in HCC patients. Another preclinical studies (123), CD28/B7 may be a predictor of the efficacy of PD-1 mAb by analyzing mouse models and samples from lung cancer patients. Meanwhile, relevant literature (124, 125) shows that microsatellite instability-high/different mismatch repair, PD-L1 expression level, the abundance of tumor infiltrating lymphocytes and tumor mutational burden can predict the efficacy of ICIs. Our retrospective study found HCC patients with alpha fetoprotein ≥ 400 ng/mL are more likely to benefit from ICIs combined with lenvatinib therapy (80). These findings indicate that the infiltration frequency and distribution pattern of tumor immune cells in HCC may affect the occurrence and development of cancer, which have certain guiding significance for distinguishing the beneficiaries of ICIs treatment and evaluating the efficacy of immunotherapy.

## Summary and Prospect

HCC is a malignant tumor with complex pathogenesis, immunogenicity and high heterogeneity (106). It is also a fortress that traditional drugs have been unable to conquer (126). Sorafenib once brought patients hope, but its overall efficacy is still not satisfactory. With the deepening understanding of the mechanism of liver immunosuppression mechanism and tumor micro-environment, immunotherapy is playing an increasingly important role in the systemic treatment of HCC. In particular, the publication of IMbrave150 (68, 69) successfully broke the monopoly of sorafenib in the first-line treatment of advanced HCC. At the same time, it also certified the potential of immune combination therapy in HCC (127). However, with the increasing researches and application of combined immunotherapy in the treatment of HCC, many problems have gradually emerged and some key problems remain to be finished. Such as how to prevent, reduce and control the AE arising from immunotherapy; how to choose the combination regimens, timing of administration and balance the relationship between side effects and efficacy; how to classify patients according to their own conditions and develop the best treatment strategy. All these questions are worthy of deep thinking. This article reviewed the development status and challenges involved in tumor ICIs and specifically elaborated the methods and advantages as well as the disadvantages of tumor immunotherapy. However, as discussed earlier, the treatment of tumor continues to face several technical hurdles. The mechanisms of tumor development and treatment still need to be further conducted in-depth research and explored. More safe and effective immunotherapy strategies for patients with HCC can be developed along with the in-depth exploration of HCC immunity and molecular pathology, so as to further improve the prognosis and quality of life of such patients.

## Author Contributions

J-HZ conceived the study. H-TL, M-JJ, and Z-JD drafted the manuscript and analyzed data. All authors have read and approved the final version to be published.

## Funding

This review is in part supported by the National Natural Science Foundation of China (82060510 and 81960308), ‘Guangxi BaGui Scholars’ Special Fund (2019AQ20), the Guangxi Natural Science Foundation (2018GXNSFBA138018, 2020GXNSFAA159022), Guangxi Key Research and Development Program (GuikeAB18126055), the Guangxi Undergraduate Training Program for Innovation and Entrepreneurship (202110598178, 202110598073, 202110598038), and High-level innovation team and outstanding scholar program in Guangxi Colleges and Universities.

## Conflict of Interest

The authors declare that the research was conducted in the absence of any commercial or financial relationships that could be construed as a potential conflict of interest.

## Publisher’s Note

All claims expressed in this article are solely those of the authors and do not necessarily represent those of their affiliated organizations, or those of the publisher, the editors and the reviewers. Any product that may be evaluated in this article, or claim that may be made by its manufacturer, is not guaranteed or endorsed by the publisher.
